# Patterns, Barriers, and Preferences of Treating Migraine Within the School Setting: A Survey Study of Students

**DOI:** 10.3390/children11111286

**Published:** 2024-10-25

**Authors:** Andrew D. Hershey, Sharon Shmuely, Alit Stark-Inbar, Yara Asmar, Alon Ironi, Eric Strong, Marielle Kabbouche

**Affiliations:** 1 Division of Neurology, Cincinnati Children’s Hospital Medical Center, Cincinnati, OH 45229, USA; andrew.hershey@cchmc.org (A.D.H.); marielle.kabbouche@cchmc.org (M.K.); 2 Department of Pediatrics, College of Medicine Cincinnati, University of Cincinnati, Cincinnati, OH 45267, USA; 3 Theranica, Netanya 4250438, Israel; sharons@theranica.com (S.S.); yaraa@theranica.com (Y.A.); aloni@theranica.com (A.I.); 4 Department of Child Neurology, Geisinger Medical Center, Danville, PA 17822, USA; estrong@geisinger.edu

**Keywords:** migraine, headache, children, adolescents, school, treatment barriers, treatment preferences, Remote Electrical Neuromodulation (REN), Nerivio

## Abstract

**Background/Objectives:** Migraine affects 10% of adolescents and children. Typical school protocols in the USA require pharmacological medications to be administered by school nurses, often resulting in treatment delays or omissions when migraine attacks occur during school hours. The Remote Electrical Neuromodulation (REN) wearable is an FDA-cleared smartphone-controlled device delivering acute and preventive treatment of migraine attacks in patients aged 8 and above, allowing safe, effective, discreet, and independent usage. **Methods:** This retrospective study (NCT06180577) evaluates treatment patterns, barriers, and preferences among school-age students. REN users < 18 years old were invited to complete an online survey. Participants signed an assent form, and their parents/legal guardians signed an informed consent form. **Results:** 332 patients aged 7–17 (15.5 ± 2.1) participated (80.4% female). After being prescribed the REN wearable, the percentage of students who treated their headaches at school increased from 78.3% to 89.8%. Most participants (65.4%) treated with either REN standalone (38.0%) or in combination with medications (27.4%). Common barriers to treatment included the need to leave class for the nurse’s office (64.2%), concerns about standing out (42.2%), and one barrier unique to REN–permission needed to use a smartphone in class (22.9%). The most common reasons given for preferring REN treatment at school are the ability to avoid going to the nurse’s office (42.5%) and to treat discreetly (39.2%). **Conclusions:** This study underscores the challenges of managing migraine at school while suggesting the importance of the REN wearable as a discreet and independently used first-line treatment for children and adolescents.

## 1. Introduction

Migraine is a complex neurological disorder and a leading cause of disabling headaches in children and adolescents [1,2]. It affects about 1 in 10 adolescents and children worldwide [3,4,5,6], with prevalence increasing throughout childhood and adolescence [5]. According to an updated Global Burden of Disease (GBD) 2021 analysis, migraine is the most disabling neurological disease among children and adolescents [7]. The impact of migraine in children and adolescents extends beyond physical discomfort and can be as severe as that of diabetes, arthritis, and cancer [8,9]. Children and adolescents with migraine report greater impairment in school, academic performance, social activities and social life, sleep issues, behavioral disturbances, and lower emotional functioning when compared to their peers, causing a negative impact on their overall quality of life [5,8,10,11,12,13]. They are also at higher risk of developing anxiety, depression, and additional pain disorders in the transition from adolescence to adulthood [14]. Yet early and effective treatment can limit disease progression and disability [15]. Managing migraine attacks in children and adolescents is therefore crucial to prevent progression into intractable disabling headache and improve long-term outcomes [14,15].

Managing migraine attacks at school poses unique challenges. In the United States, school policies typically require pharmacological medications to be administered by school nurses, forcing students to leave class and thus disrupting their academic routine [16,17]. This interruption and lack of independence often leads to missed or delayed treatments [16,17]. The need to step out of class can further increase the stigma around migraine. The stigma of migraine, which is evident across a wide range of ages and countries, is itself associated with negative outcomes, higher disability, and reduced quality of life [11,18,19,20]. Therefore, non-pharmacological treatments that can be used independently and discreetly during class are essential.

The Remote Electrical Neuromodulation (REN) wearable device addresses these challenges. This FDA-cleared, smartphone-controlled, prescribed device offers a drug-free therapy for acute and preventive migraine treatment in individuals aged 8 and older [21,22]. It enables discreet and independent disease management, beyond traditional pills and injections. Studies indicate REN is safe, effective, and well tolerated in acute and preventive migraine treatment in adolescents (12–17 years old) [23,24,25,26] and children (6–12 years old) [27].

This study explores the barriers and preferences for treating migraine headaches within school settings. It seeks to shed light on the potential of REN to improve migraine management and the well-being of children and adolescents living with migraine through the greater autonomy and convenience it delivers.

## 2. Methods

### 2.1. Ethics and Study Enrollment

The study was conducted according to the guidelines of the Declaration of Helsinki, was approved by the WCG Institutional Review Board (WCG 20233847), and was registered at clinicaltrials.gov (NCT06180577). Informed consent was obtained from all participants and their legal guardians.

### 2.2. Study Design and Participants

The study was based on a retrospective, cross-sectional survey that took place between 26 December 2023 and 10 March 2024.

**Figure 1:**
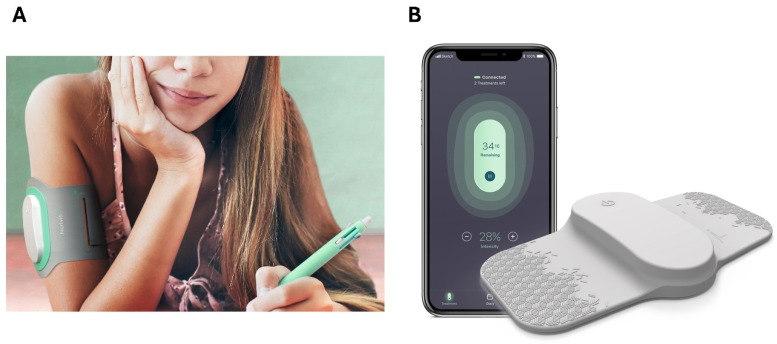
The REN wearable device. (**A**) A teenager wearing the REN device on her upper arm. Patients can go about their day, study, attend class, socialize, etc., while wearing the device. (**B**) The REN wearable is composed of the Nerivio^®^ device, an armband with varying sizes, and the Nerivio^®^ smartphone app used to start treatment, adjust treatment intensity, and manage migraine treatment. REN, remote electrical neuromodulation.

All study participants were children and adolescents aged 17 years (inclusive) or younger who were prescribed the REN wearable (Figure 1) by healthcare providers in the USA for the treatment of their migraine attacks. Following prescription, a specialty pharmacy collects co-insurance fees and dispenses the REN device (Nerivio, Bridgewater, NJ, USA) to the patient’s home. To use the device, patients need to download the Nerivio^®^ app to their smartphone, create a user account, and connect the device to their account via Bluetooth. To perform a treatment, the patient should attach the device to the back of their upper arm with the electrodes touching the skin, seal the device with an armband, and press the ON button. Patients are instructed to use the app to increase the stimulation intensity to a level that is strong, well felt, and yet not painful. This is completed by pressing the +/− signs on the app. Each treatment lasts for 45 min, during which the patient can go about their day. The device can be worn discreetly, hidden under a shirt sleeve. After the treatment, the device is stored until the next treatment. Treatment instructions for acute treatment are to begin treatment as soon as possible at the first sign of a migraine attack, and, for migraine prevention, to use every other day. The Nerivio box comes with a startup guide, and there are many videos explaining how to use the device. Patients who were prescribed Nerivio could decide whether to use the REN device at all, as with any other treatment, and specifically whether to use it on school premises.

REN patients under the age of 18 were invited by email or in-app notification to be screened for enrollment in this study. Eligibility was verified via an electronic screening questionnaire prior to enrollment and included (1) younger than 18 years of age; (2) treated with the device at least once; (3) attended an in-person (rather than online) elementary, middle, or high school when first prescribed the device. Patients who were deemed eligible were offered the opportunity to participate in the study. Those who were willing to participate were directed to an electronic informed consent form (e-ICF) to be signed by their parent/legal guardian. After electronic parental consent was obtained, an electronic assent form was provided for the patient to sign. Once both electronic consent forms were signed and the participant was enrolled, they were directed to the study survey. Data regarding treatment patterns were pulled from the REN data server. Note that participants were prescribed the REN wearable over a range of treatment durations prior to study enrollment, therefore creating different treatment duration windows. Participants were compensated for their study participation time.

### 2.3. Study Instrument

The study was conducted as an electronic survey comprised of 25 questions divided into the following sections: (1) demographics and medical history associated with migraine; (2) preferences and barriers for treating migraine attacks in a school setting; (3) preferences and barriers for treating migraine attacks with the REN wearable in a school setting; and (4) acute and preventive medication patterns. These questions were developed using multiple rounds of the Delphi method by Headache Medicine experts that focused on the treatment of headaches in children and adolescents. Demographic and medical history questions regarding the frequency of attacks/treatments and prescription type were single-select questions. Questions regarding treatment barriers and preferences were multiple-choice questions.

### 2.4. Outcome Measures

#### 2.4.1. Primary Outcome Measure

The percent of students who prefer treating migraine headaches in the school setting with the REN wearable over other options.

#### 2.4.2. Secondary Outcome Measures

(1) Distribution of treatment barriers by students who need to treat their headaches while at school.
(2) Distribution of the types of treatments used by students at school to treat migraine headaches prior to being prescribed the REN wearable (pharmaceutical agents or not treated at all) who subsequently discontinued treatment, reflecting convergence to using REN at school.

### 2.5. Sample Size

The sample size for this study was calculated based on the prevalence of migraine in 10% of school-aged children in the United States, which corresponds to approximately 5 million individuals. Using a confidence interval of 90% and a margin of error of 5%, the required sample size was 271 participants.

### 2.6. Data Analysis

Data analysis was conducted using Microsoft Excel software (version 365). Descriptive statistics were used to summarize the demographic and clinical characteristics of the participants, including frequencies and percentages for categorical variables, and means with standard deviations were used for continuous variables. For the primary outcome measure, the percentage of participants treated with the REN wearable alone or in combination with other medications was calculated related to other treatment options; those who were not treated at school were not included in this calculation. For the secondary outcome measures, the distribution of treatment barriers reported by participants was analyzed using percentages and was presented via a bar graph. The distribution of treatment types used by patients prior to being prescribed REN was also calculated and represented by percentages. While the primary and secondary outcomes were descriptive, we conducted statistical tests to compare treatment behaviors before being prescribed REN vs. after. Statistical testing was conducted with two-tailed Chi-square tests, and a *p*-value < 0.05 was considered statistically significant.

## 3. Results

### 3.1. Disposition

**Figure 2:**
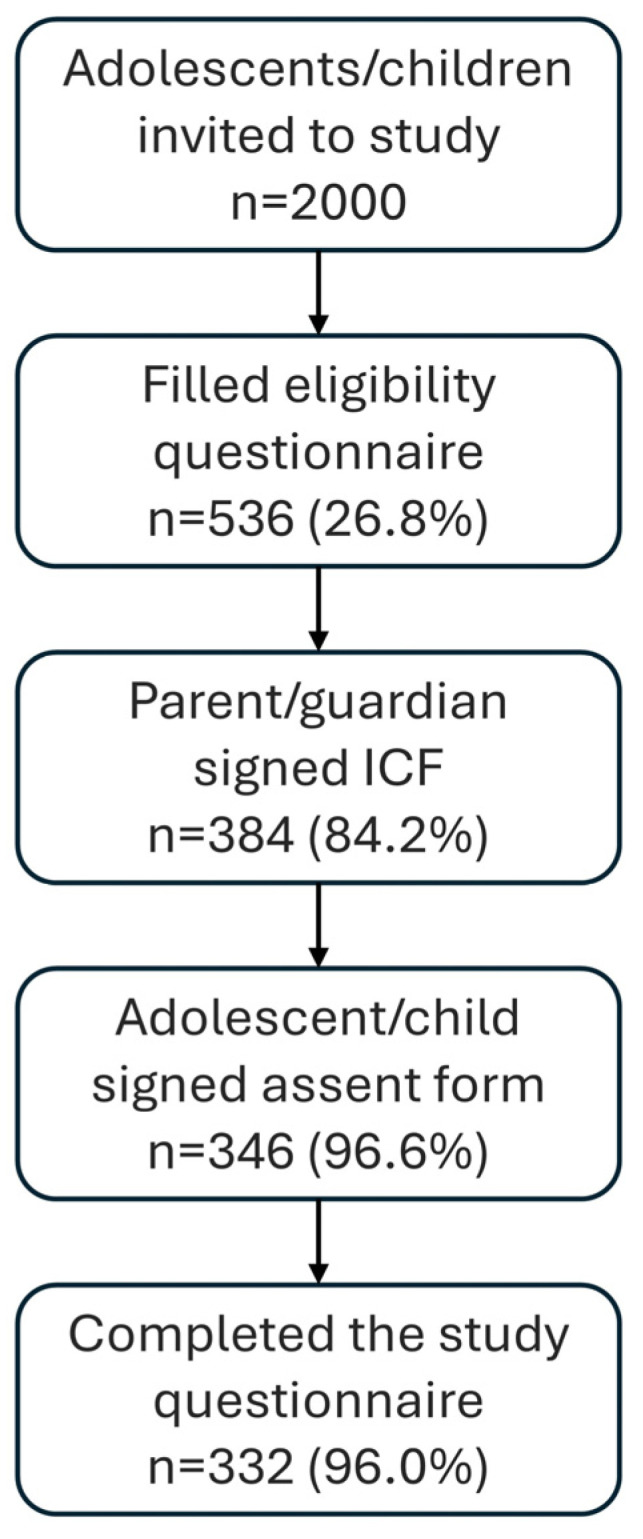
Study disposition. Three hundred thirty-two adolescents and children participated in the retrospective survey study.

Figure 2 provides a flow chart of the study participants. Two thousand migraine patients under the age of 18, who treated with the REN wearable device at least once, were approached. Five hundred and thirty-six REN users filled out the electronic eligibility questionnaire (26.8%). Of these, 456 (85.1%) were found to be eligible. Of the eligible users, 384 (84.2%) parents/legal guardians signed the e-ICF and 346 (96.6%) of their children signed the assent form. A sample of 332 (96.0%) participants with signed forms completed the study questionnaire. Data from all 332 participants who completed the study questionnaire were included in the analysis.

### 3.2. Participant Characteristics

Participants were 7–17 years old (mean 15.5 ± SD 2.1), predominantly female (80.4%), and white (82.2%). Most participants were either in high school (68.7%) or middle school (23.5%), and the majority attended public school (78.9%). More than half of the participants (59.9%) were prescribed REN for prevention or dual-use (i.e., acute and prevention), while the remaining (40.1%) were prescribed REN for acute treatment only. The number of headaches per month varied between patients, with most participants reporting a frequency of headaches that reflects chronic migraine (48.2% reported 15 or more headaches a month) or high-episodic migraine (25.0% reported 8–14 headaches a month). Most participants (82.5%) stopped using one or more types of acute medications before starting REN, and 57.8% stopped one or more types of preventive medications before starting REN. On average, participants conducted 33.4 ± 29.8 REN treatments, with 64.8% of the participants treating at least 18 times. Table 1 shows demographic and clinical characteristics.

### 3.3. Treatment Prior to Being Prescribed REN

**Figure 3:**
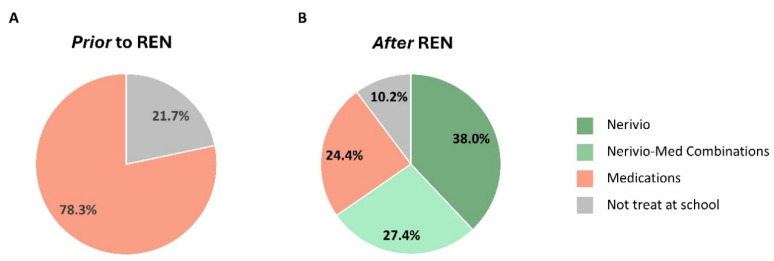
Distribution of treatment types used by participants to treat migraine headaches while they are at school prior to being prescribed REN (**A**) and after being prescribed REN (**B**).

The distribution of treatment types used by participants to treat their headaches while at school prior to being prescribed REN was analyzed. Here, 78.3% reported using medications (49.7% used prescribed medications, 28.6% used over-the-counter medications), and 21.7% did not treat at all while at school. No one reported using another device before being prescribed REN (Figure 3A).

**Figure 4:**
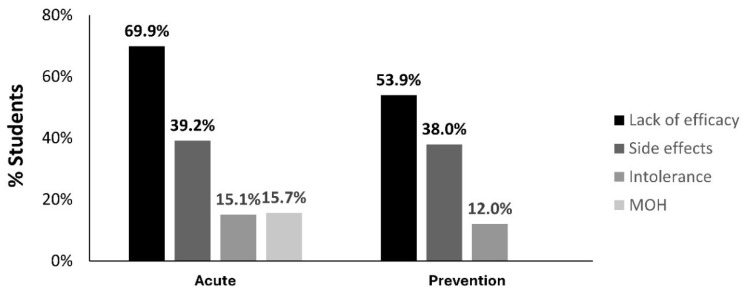
Reasons for discontinuing previous acute and preventive treatments for migraine, as measured by the percentage of participants reporting each reported reason.

Participants who discontinued previous migraine treatments before being prescribed the REN wearable reported one or more causes for discontinuation, mostly the lack of efficacy (69.9% with acute medications; 53.9% with prevention medications), side effects (39.2% with acute medications; 38.0% with prevention medications), intolerance to the medication (15.1% with acute medications; 12.0% with prevention medications), or medication overuse headache (MOH, 15.7%, only with acute medications) (see Figure 4). Additionally, nearly half of the study participants (49.7%) reported that it took over 30 min to start treating their headaches while at school.

### 3.4. Barriers to Treatment at School

**Figure 5:**
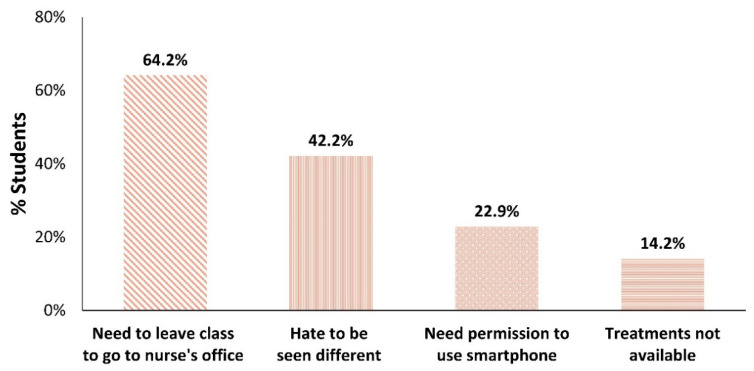
Distribution of participants’ barriers to treatment at school. The same bar patterns are used in both Figure 3 and Figure 4 to reflect converging barriers and preferences.

Participants were asked about barriers to treating migraine headaches within the school setting, possibly indicating multiple barriers (Figure 5). The most common treatment barrier, reported by nearly two-thirds of the students (64.2%), was the need to leave class to go to the nurse’s office. The following most common treatment barriers included hating to be seen as different (42.2%), the need for permission to use a smartphone in class (22.9%), and not having treatments available to them (14.2%).

### 3.5. Treatment Preferences at School

Participants were asked about their treatment patterns while at school once they had been prescribed the REN wearable (Figure 3B). The portion of participants who do not treat at all while at school was significantly (*p* < 0.0001) reduced by more than half, from 21.7% (Figure 3A, gray) to 10.2% (Figure 3B, gray). The portion of participants who use pharmacological medications while at school was significantly (*p* < 0.0001) reduced by nearly two-thirds, from 78.3% (Figure 3A, orange) to 24.4% (Figure 3B, orange). Of the remaining participants, 27.4% combined REN with medications (reflecting either combination therapy for the same attack or alternating between therapies over different attacks; Figure 3B, light green) or used REN as a standalone treatment for all migraine headaches while at school (38.0%; Figure 3B, dark green). Taken together, once been prescribed the REN wearable, 65.4% of the students reported treating with REN either as a standalone therapy or in combination with other medications while at school.

**Figure 6:**
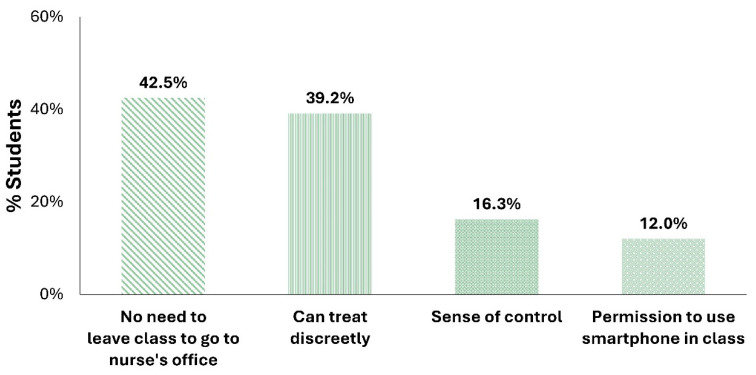
Distribution of participants’ preferences for treating migraine headaches with REN while at school. The same bar patterns are used in both Figure 5 and Figure 6 to reflect converging barriers and preferences.

When asked about the reasons for this preference, participants could have provided one or more reasons (Figure 6). Participants reported a preference for the REN wearable mainly because they were able to remain in class (42.5%). Other leading reasons for preferring REN included the ability to treat discreetly (39.2%), REN giving them a sense of control over their disease (16.3%), and REN gaining them permission to use their smartphone in class (12.0%).

Note that the top two barriers to treating migraine headaches while at school—the need to leave class to go to the nurse’s office and hating to seem different (Figure 5)—were resolved through treatment with the REN wearable which can be used in class without the need to go to the nurse’s office, and which also offers a discreet option for treatment. Given that the REN wearable is fully controlled by a smartphone app, it poses a unique barrier that some patients face, depending on their school regulations: the need to get permission to use a smartphone in class. Some participants report gaining permission to use their smartphone in class in order to treat with the REN wearable as an important advantage.

## 4. Discussion

This study highlights the challenges children and adolescents face in treating migraine headaches at school, emphasizing key barriers such as lack of access to treatment, inconvenience, and social stigma. The REN wearable offers advantages over traditional pharmacological treatments in terms of accessibility, reducing learning disruptions, reducing social stigma, and fostering autonomy. The findings suggest that REN improves treatment adherence at school, reducing the portion of students who either avoid treatment or rely solely on pharmacological medications. Given the need for timely intervention and adherence to treatment especially while at school, this study has immediate clinical implications for school-aged students living with migraine.

### 4.1. Migraine Treatment at School

Children and adolescents encounter unique challenges in treating migraine at school [10]. Before having been prescribed the REN wearable, more than 1 out of 5 study participants (21.7%) did not treat their migraine headaches while at school, indicating a gap in effective migraine management among students. Once having access to the REN wearable, this treatment pattern shifted, with only 1 out of 10 avoiding treatment while at school. Thus, having an accessible treatment within arms-reach at school provided a treatment option that was adopted by half of the students who would otherwise have avoided treatment at school. Moreover, the portion of students using only pharmacological medications to treat their headaches while at school was reduced three-fold (from 78.3% to 24.4%) once they could use REN. Nearly two-thirds of the participants use REN at school, with 38.0% using it as a standalone treatment for their attacks while at school and 27.4% combining or alternating between REN and medications.

A notable aspect of the findings is the alignment between participants’ main barriers to treating headaches due to migraine at school and the primary reasons for their preference for treating migraine with the REN wearable at school. Students marked three main barriers to treating migraine headaches at school. The most dominant barrier, reported by 64.2% of participants, was the need to leave class to go to the nurse’s office to receive treatment. Correspondingly, a major preference for REN, as 42.5% of participants noted, is the ability to be treated in the classroom without going to the nurse’s office. This preference directly mitigates disrupting class activities and the inconvenience of leaving the classroom and going to the nurse’s office to receive medication [16,17].

The second treatment barrier is the stigma of seeming different, as mentioned by 42.5% of participants. In parallel, 39.2% of participants preferred treatment with REN at school because it allowed for discreet treatment. Migraine has a severe impact on school-aged children and adolescents, negatively affecting not only their academic performance, but also their social activities, emotional functioning, and overall quality of life [5,8,10,11,12]. Previous research suggests that non-pharmacological treatments can reduce the psychosocial burden of migraine in people under the age of 18 [8,26], highlighting additional benefits to students beyond the previously reported physiological efficacy and functional benefits of REN [23,24,25,26,27].

The alignment between the most common barriers for migraine treatment at school and the preference for treating with REN underscores the potential of REN to address critical challenges specifically faced by students in managing migraine at school. REN can significantly enhance the management of migraine headaches in school settings, providing a more integrated, user-friendly treatment option for young migraine sufferers.

However, REN poses a new barrier, ranked as the third most common by 22.9% of the participants. To use REN, many students in the USA need permission to use their smartphones in class, as not all schools allow students to keep their smartphones accessible during class. Despite this, 12.0% of the participants highlighted the benefit of being allowed to have their smartphone nearby, as it is necessary to operate the REN device. The gap between the need for permission and the benefit of having a smartphone nearby could result from the fact that in some USA schools, there is no issue (and therefore no clear benefit) in having smartphones in class. Students who still face this restriction may solve the issue by sending a letter to school from their healthcare provider, explaining their migraine diagnosis and their REN wearable treatment, which requires smartphone control. The authors of this paper have had great success in gaining such permission when patients are provided with such a letter early on, at the time of their appointment in the clinic.

### 4.2. Study Limitations

The main study limitation is that all participants were recruited from the REN database, creating a potential bias favoring REN over other treatments. That said, in almost every study of treatment interventions, whether a controlled trial or a real-world study, participants are typically those who are not fully satisfied with their existing therapies and try more novel treatments. Moreover, in the current study, there was no recruitment bias favoring frequent REN users or those who were treated with REN within school settings, as any student treated with REN at least once in any location (home, school, elsewhere) was eligible to participate in the study.

### 4.3. Clinical Implications and Future Research

The results have immediate implications for clinical practice. First, the non-pharmacological nature of REN alleviates parental concerns relating to drug safety and medication overuse, providing a safe and effective long-term drug-free treatment for children and adolescents that can be used both at home and at school [26,28]. Second, the results highlight the need for prescribing REN as a first-line treatment for children and adolescents who attend grade school, bypassing the often burdensome step therapy required by many insurance companies. A recent survey shows that most migraine patients in the USA (93%) are subject to step therapy prior to approving a treatment prescribed by their healthcare provider [29]. The current study shows that most young participants discontinued one or more acute (82.5%) or preventive (57.8%) medications before being prescribed the REN wearable. Thus, offering REN as a first-line treatment can save the need to unsuccessfully use and stop traditional treatments that lack efficacy, have side effects, are intolerable, or lead to medication overuse headaches. Ineffective treatments have a negative impact on patients’ physical and mental well-being, hindering their ability to attend school and socialize, increasing the risk of disease progression and chronification [29].

Future research and advocacy should focus on increasing awareness to the benefits of REN among school administrators, school nurses, and healthcare providers. Integrating REN into standard school health practices, where traditional pharmacological treatments pose significant challenges [16,17], could improve migraine management in students, offering a user-friendly, effective alternative to traditional medications.

## 5. Conclusions

This study supports the hypothesis that students often refrain from treating their migraine headaches at school due to the need to leave class and the social discomfort in asking for medications. The REN wearable offers a drug-free, discrete, and effective treatment that can be self-administered without the need to leave class or face stigma. By addressing the key barriers associated with migraine and the treatment of migraine at school, REN has the potential to transform migraine management in school-aged children and adolescents. REN should therefore be evaluated as a first-line treatment option covered by insurance for school-aged students living with migraine.

## Figures and Tables

**Table 1:** Demographic and clinical characteristics.

		% (n)
Age (years)	Overall (Average ± SD)	15.47 ± 2.12
	17	29.8% (99)
	16	20.2% (67)
	15	13.0% (43)
	14	18.4% (61)
	13	7.8% (26)
	12	2.1% (7)
	Under 12	8.7% (29)
Gender	Female	80.4% (267)
	Male	14.2% (47)
	Non-Binary	3.6% (12)
	Transgender	0.9% (3)
	Other/would rather not specify	0.9% (3)
Race	White	82.2% (273)
	Black or African American	4.2% (14)
	Hispanic or Latino	4.8% (16)
	Asian or Asian American	3.6% (12)
	American Indian or Alaska Native	0.3% (1)
	Hawaiian or Pacific Islander	0.0% (0)
	Other	4.8% (16)
School	High School	68.7% (228)
	Middle School	23.5% (78)
	Elementary School	7.8% (26)
REN Prescription Type	Dual Use	50.3% (167)
	Acute	40.1% (133)
	Prevention	9.6% (32)
Headache Frequency	15 times per month or more	48.2% (160)
	8–14 times per month	25.0% (83)
	4–7 times per month	17.2% (57)
	1–3 times per month	9.6% (32)
Number of acute medications quit before REN Rx	No acute medications stopped	17.5% (58)
	1 acute medication stopped	36.4% (121)
	2 acute medications stopped	24.4% (81)
	3 or more acute medications stopped	21.7% (72)
Number of preventive medications quit before REN Rx	No preventive medications stopped	42.2% (140)
	1 preventive medication stopped	41.3% (137)
	2 preventive medications stopped	9.3% (31)
	3 or more preventive medications stopped	7.2% (17)
REN Treatments performed prior to the study	Above 18 treatments	64.8% (215)
	13–18 treatments	15.1% (50)
	7–12 treatments	8.7% (29)
	1–6 treatments	11.4% (38)

## Data Availability

The data presented in this study are available on request from the corresponding author (the data are not publicly available due to privacy or ethical restrictions).
